# *Blastomyces dermatitidis* Yeast Lysate Antigen Combinations: Antibody Detection in Dogs with Blastomycosis

**DOI:** 10.1155/2013/940126

**Published:** 2013-10-08

**Authors:** Alex R. Boyd, Jamie L. VanDyke, Gene M. Scalarone

**Affiliations:** Department of Biological Sciences, Idaho State University, P.O. Box 8007 921 S. 8th Avenue, Pocatello, ID 83209, USA

## Abstract

The systemic fungal infection, blastomycosis, which infects both humans and animals has presented a diagnostic challenge for clinicians for many years. The aim of this study was to evaluate the diagnostic sensitivity of *Blastomyces dermatitidis* yeast lysate antigens with respect to antibody detection in dogs with blastomycosis. Lysate antigens were prepared from *B. dermatitidis* isolates T-58 and T-66 (dogs, Tennessee) and WI-R and WI-J (dogs, Wisconsin). Based on results obtained from a preliminary comparative study, five combinations of these isolates and one individual isolate were tested against 92 serum specimens from dogs with culture-proven or histologically-confirmed blastomycosis, using the indirect enzyme-linked immunosorbent assay (ELISA). Mean absorbance values obtained from the sera ranged from 0.905 with the individual T-58 antigen to 1.760 using an antigen combination (T-58 + T-66 + WI-R). All of the 6 antigenic preparations were able to detect antibody in the serum specimens, but the antigen combinations detected antibody to a higher degree than the individual antigen. This study provides evidence that combinations of the yeast lysate reagents seem to be more efficacious for antibody detection in dog sera, but our laboratory is continuing to evaluate antigen lysate combinations for detection of antibodies in blastomycosis.

## 1. Introduction

Blastomycosis, a systemic fungal infection of humans and animals, is produced by the dimorphic fungal organism *Blastomyces dermatitidis. *The infection is initiated by the inhalation of spores produced by the filamentous phase of the fungus. The organism exists in this stage in nature or in the laboratory at 25°C and has the ability to convert to the yeast phase at 37°C in the lungs of the infected host. The disease may be self-resolving or it may exist as an acute or chronic state in the pulmonary tissue. If the disease is untreated while in the lungs, it may become invasive and disseminate to other organs and possibly to the central nervous system where fatal meningitis may develop [1–5]. Blastomycosis, as well as other systemic mycoses, are termed “emerging fungal threats” since they not only infect persons with normal immune systems but are also a cause for concern in immunocompromised individuals [3, 4].

The geographic distribution of blastomycosis has been associated with southeastern and south-central states that border the Ohio and Mississippi Rivers and upper midwestern states including areas in Wisconsin and Minnesota, which are highly endemic for the disease [6, 7].

Due to the increase in systemic fungal diseases, researchers have begun to devote more attention to developing ways of diagnosing, preventing, and treating these mycoses [8–17]. For the past several years the thrust of research in our laboratory has been associated with studies on various strains of *B. dermatitidis *from human, animal, or environmental specimens from many geographical locations in an effort to better understand the immunobiology of the organism [18–27]. Diagnosis of the disease has presented major problems. In some instances, culturing or histopathological examination may be beneficial, but in some patients these methods may not yield the desired results. This has led to more and more research being done to improve immunological assays which tend to provide a more rapid diagnosis, but we, as well as many other investigators, recognize that problems still exist with regard to the sensitivity and specificity of immunoassays. 

Our current studies are aimed at evaluating combinations of the various *B. dermatitidis *yeast lysate antigens for the detection of antibodies in serum specimens from dogs with blastomycosis.

## 2. Materials and Methods

### 2.1. Antigens

Yeast phase lysate reagents (T-58, dog Tennessee; T-66, dog Tennessee; WI-R, dog Wisconsin; and WI-J, dog Wisconsin) were prepared by a method similar to one that was previously used for the production of antigen from *Histoplasma capsulatum* [28–30] and modified in our laboratory for *B. dermatitidis* lysate antigen production [23]. The yeast phase cells were grown for 7 days at 37°C in a chemically defined medium (glucose, 10.0 g; potassium phosphate monobasic, 1.5 g; calcium chloride dehydrate, 0.15 g; magnesium sulfate, 0.5 g; ammonium sulfate, 2.0 g; L-asparagine, 2.0 g; L-cysteine, 0.2 g; and pH adjusted to 6.2 with 5 N sodium hydroxide) in an incubator shaker, harvested by centrifugation (700 ×g; 5 min) followed by washing with distilled water, resuspended in distilled water, and then allowed to lyse for 7 days at 37°C in water with shaking. The preparations were centrifuged, filter sterilized, merthiolate added (1 : 10,000), and stored at 4°C. Protein determinations were performed on the lysates using the BCA protein assay kit (Pierce Chemical Company, Rockford, IL, USA), and dilutions of the antigenic reagents used in the ELISA assays were based on protein concentration. Combinations of the above four antigenic reagents as well as T-58 (not combined with others) were used for antibody detection. A previous preliminary comparative evaluation was performed [27] using a number of individual and combinations of the above lysate preparations to assess their ability to detect antibodies in 24 sera from dogs with blastomycosis. This study indicated that 6 of the preparations showed the greatest degree of sensitivity. Therefore, this present study, with a much greater number of serum specimens, was initiated to further evaluate the 6 optimal lysate reagents (T-58 + T-66 + WI-R; T-66 + WI-R; T-58 + WI-J; T-66 + WI-R + WI-J; T-58 + T-66, and the one individual antigen T-58) from the earlier study for antibody detection in 92 sera from dogs with diagnosed blastomycosis but with varying amount of antibody in the specimens.

### 2.2. Serum Specimens

Ninety-two serum specimens from dogs with diagnosed blastomycosis were provided by Dr. A. M. Legendre (University of Tennessee College of Veterinary Medicine, Knoxville, TN, USA). Negative (normal) sera were not included in this study since we were interested in comparing reactivity and not correcting for background with negative controls.

### 2.3. Enzyme-Linked Immunosorbent Assay (ELISA)

The ability of each of the 6 (individual or combination preparations) yeast lysate reagents to detect antibody in the above serum specimens was determined using the indirect enzyme-linked immunosorbent assay (ELISA). Each lysate antigen was diluted (2000 ng of protein/mL) in a carbonate-bicarbonate coating buffer (pH 9.6; equal amounts of each lysate were admixed in preparing the combinations and 2000 ng of protein/mL of the individual T-58 antigen resulting in 200 ng total protein/100 uL in each well) and then added to triplicate wells (100 uL) of a NUNC 96-well microplate (Fisher-Thermo). The plates were then incubated overnight at 4°C in a humid chamber followed by washing three times with phosphate buffered saline containing 0.15% Tween 20 (PBS-T). The serum specimens (1 : 2500 dilution; 100 uL) were added to the microplate wells and incubated for 30 min at 37°C in a humid chamber. Following this incubation the wells were washed as above and 100 uL of goat anti-dog IgG (H & L) peroxidase conjugate (Kirkegaard and Perry, Gaithersburg, MD, USA) was added to each well and incubated for 30 min at 37°C. The plates were again washed as above and 100 uL of TMB peroxidase substrate (Pierce/Fisher-Thermo) was added to each well and incubated for approximately 2 min at room temperature. The reaction was stopped by the addition of sulfuric acid and the absorbance read at 450 nm using a BIO-RAD 2550 EIA reader. 

## 3. Results/Discussion

**Figure 1:**
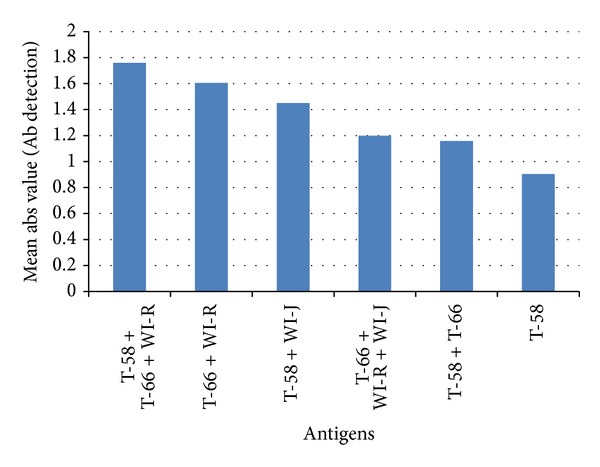
Comparison of the 6 *B. dermatitidis *yeast lysate antigens for the detection of antibodies in serum specimens from 92 dogs with diagnosed blastomycosis.

The mean absorbance values of the six *B. dermatitidis* lysate antigens, when used in the ELISA to detect antibodies in 92 dog sera, are shown in Figure 1. The five reagent combinations exhibited mean absorbance values greater than one, ranging from 1.158 to 1.760, while the single antigenic reagent (T-58) exhibited a mean absorbance value of 0.905. The most reactive reagent was T-58 + T-66 + WI-R, a mixture of two southern isolates and one northern isolate. All of the reagents were able to detect antibodies against blastomycosis with the optimal reagent detecting antibody at twice the rate of the single antigen. 

The sensitivity of the 6 lysate preparations, when evaluated on their ability to detect antibody at mean absorbance values ranging from 0.400 to 2.800, is shown in Table 1. The sensitivity percentages with the optimal lysate antigen (T-58 + T-66 + WI-R) range from 100% at the 0.400−0.800 absorbance range to 42% at the highest absorbance value (2.101–2.800). Sensitivity percentages ranged from 25% to 0% with the other 5 lysates at this highest absorbance value level.

Most previous studies have evaluated only single antigens for the detection of antibodies in blastomycosis. However, one previous study tested 14 antigens or antigen combinations against 24 dog sera to determine sensitivity using the ELISA [27]. In this study, we used the 6 most reactive reagents from that study to further examine sensitivity in this comparative assay.

## 4. Conclusion

This study indicates that certain combinations of antigens are preferable as immunodiagnostic reagents for antibody detection of *B. dermatitidis *in sera from dogs. The T-58 + T-66 + WI-R antigen combination is a promising candidate for future studies based on this comparative study. Therefore, our laboratory is continuing studies in an effort to develop a reagent that will aid in the reliable immunodiagnosis of blastomycosis in humans and animals.

## Figures and Tables

**Table 1a:** (a)

Absorbance range	T-58 + T-66 + WI-R	T-66 + WI-R	T-58 + WI-J	T-66 + WI-J + WI-R	T-58 + T-66	T-58
0.400–0.800	4	2	12	14	22	47
0.801–1.200	12	20	25	35	29	21
1.201–1.600	20	22	15	28	23	15
1.601–2.000	17	25	20	12	17	9
2.010–2.400	32	22	16	3	1	0
2.401–2.800	7	1	4	0	0	0

**Table 1b:** (b)

Absorbance range	T-58 + T-66 + WI-R	T-66 + WI-R	T-58 + WI-J	T-66 + WI-J + WI-R	T-58 + T-66	T-58
>0.4	100%	100%	100%	100%	100%	100%
>0.8	96%	98%	87%	85%	76%	49%
>1.2	83%	76%	60%	47%	45%	26%
>1.6	61%	52%	44%	16%	20%	10%
>2.0	42%	25%	22%	37%	19%	0%
